# Effects of Different Livestock Grazing on Foliar Fungal Diseases in an Alpine Grassland on the Qinghai–Tibet Plateau

**DOI:** 10.3390/jof9090949

**Published:** 2023-09-20

**Authors:** Zhen Tian, Wenjie Li, Yixin Kou, Xin Dong, Huining Liu, Xiaoxia Yang, Quanmin Dong, Tao Chen

**Affiliations:** 1 State Key Laboratory of Herbage Improvement and Grassland Agro-Ecosystems, Center for Grassland Microbiome, College of Pastoral Agricultural Science and Technology, Lanzhou University, Lanzhou 730000, China; tianzh20@lzu.edu.cn (Z.T.); liwenjie19@lzu.edu.cn (W.L.); kouyx20@lzu.edu.cn (Y.K.); dongx21@lzu.edu.cn (X.D.); liuhn21@lzu.edu.cn (H.L.); 2 Qinghai Provincial Key Laboratory of Adaptive Management on Alpine Grassland, Qinghai Academy of Animal Science and Veterinary Medicine, Qinghai University, Xining 810016, China; xxyang@qhu.edu.cn (X.Y.); qmdong@qhu.edu.cn (Q.D.)

**Keywords:** alpine grasslands, community pathogen load, fungal diseases, herbivores, species richness

## Abstract

In grassland ecosystems, the occurrence and transmission of foliar fungal diseases are largely dependent on grazing by large herbivores. However, whether herbivores that have different body sizes differentially impact foliar fungal diseases remains largely unexplored. Thus, we conducted an 8-year grazing experiment in an alpine grassland on the Qinghai–Tibet Plateau in China and tested how different types of livestock (sheep (*Ovis aries*), yak (*Bos grunniens*), or both)) affected foliar fungal diseases at the levels of both plant population and community. At the population level, grazing by a single species (yak or sheep) or mixed species (sheep and yak) significantly decreased the severity of eight leaf spot diseases. Similarly, at the community level, both single species (yak or sheep) and mixed grazing by both sheep and yak significantly decreased the community pathogen load. However, we did not find a significant difference in the community pathogen load among different types of livestock. These results suggest that grazing by large herbivores, independently of livestock type, consistently decreased the prevalence of foliar fungal diseases at both the plant population and community levels. We suggest that moderate grazing by sheep or yak is effective to control the occurrence of foliar fungal diseases in alpine grasslands. This study advances our knowledge of the interface between disease ecology, large herbivores, and grassland science.

## 1. Introduction

As an important component of ecosystems, foliar fungal pathogens play a key role in maintaining plant diversity and stabilizing ecosystem structure and function [1,2]. Both empirical studies and meta-analyses have indicated that fungal pathogens can promote the coexistence of different plant species and regulate the diversity–productivity relationship through negative density dependence and growth–defense trade-offs [1,3,4,5,6]. However, alternatively, foliar fungal pathogens can reduce photosynthesis, cause losses of plant biomass, and even kill host plants and cause severe die-off and extinction of host species in certain cases, posing serious threats to the structure and functioning of ecosystems [7,8,9]. Therefore, understanding the occurrence and transmission of foliar fungal diseases and their interactions with host plants is important for the health of ecosystems.

Livestock grazing is one of the most intensive human activities in grasslands and is a major driver of the structure and function in these ecosystems [10,11,12]. Therefore, the occurrence and severity of foliar fungal diseases in grassland ecosystems largely depend on grazing by livestock [13,14,15]. There are two potential mechanisms to explain how grazing could affect foliar fungal diseases. First, grazing could directly increase, by producing wounds, or decrease, by removing pathogens, the transmission of disease through defoliation [16,17,18]. Second, many studies have indicated that alterations in plant diversity and community composition can change the transmission and prevalence of diseases [19,20,21]. Thus, grazing can indirectly affect disease severity by altering plant diversity and community composition [13,21]. For example, when grazing livestock preferentially feed on disease-susceptible host plants, they can remove pathogens and increase the abundance of non-susceptible host plants, thereby decreasing the community pathogen load [13,22].

Grazing effects on grassland ecosystems are highly dependent on the intensity of grazing, because the increasing demand for livestock products has driven a global increase in stocking rates [23,24]. Therefore, several studies have evaluated how grazing intensity affects foliar fungal diseases [13,25,26]. However, most natural grasslands are grazed simultaneously by different herbivores, such as cattle (*Bovine*) and sheep (*Ovis aries*) [27]. Different livestock could have differential direct effects on foliar fungal diseases, since different types of grazers may vary in their grazing behavior, such as in diet selectivity or livestock characteristics such as body size [28,29,30]. For example, selective feeding on infected plant individuals may directly reduce plant diseases by eliminating the source of infection [25]. In addition, previous studies indicated that large livestock, such as cattle, can increase plant diversity, because cattle usually consume tall grasses that are dominant, whereas small livestock, such as sheep and goats, can decrease plant diversity because they prefer to graze on low-growing subordinate species, such as forbs and semi-shrubs [29,31,32]. Therefore, different livestock may indirectly affect foliar fungal diseases differentially by altering plant diversity and community composition. However, whether different livestock have differential impacts on foliar fungal diseases remains largely unexplored.

In this study, we used an 8-year field-manipulated grazing experiment, including livestock grazing by a single species (yak or sheep) and mixed species (sheep and yak), to evaluate the effects of different livestock on foliar fungal diseases of both individual host plant species (i.e., population level) and plant communities (i.e., community level). To achieve this, we quantified all foliar fungal diseases in all plots that were subjected to different grazing treatments and calculated the disease severity of individual host plant species. We also recorded the vegetation characteristics, such as total plant coverage and species richness, and calculated the community pathogen load. In particular, we tested the following hypotheses: (i) grazing by a single species (yak or sheep) or mixed species (sheep and yak) will decrease the occurrence of foliar fungal diseases; and (ii) grazing by yak will cause more pronounced reductions in the occurrence of foliar fungal diseases than grazing by sheep.

## 2. Materials and Methods

### 2.1. Study Site

Our study was conducted at the Platform of the Adaptive Management of the Alpine Grassland-Livestock System on the Qinghai–Tibet Plateau of Qinghai University, Haiyan County, Qinghai Province, China (36°40′~38°40′ N, 100°20′~101°30′ E; 3000–3100 m a.s.l.). This area is located in the eastern part of the Qinghai–Tibet Plateau and has a plateau continental climate. The mean annual temperature is 1.5 °C, with the lowest monthly average of −24.8 °C in January and the highest monthly average of 12.5 °C in August. The mean annual precipitation is 330–370 mm, of which 60–80% occurs during the growing season (from May to September) [33,34]. The soil is classified as a clay loam. The grassland vegetation is a typical alpine meadow dominated by the *Cyperaceae* species *Kobresia humilis* and *Carex aridula* [33]. Grazing by livestock (primarily sheep and yak) is a common practice in this area, and most of the grasslands are moderately to heavily grazed [33,34].

### 2.2. Grazing Experimental Design

A long-term grazing experiment was initiated in 2014 with a completely randomized block design. Sheep and yak were used to establish the grazing experiment. Three blocks (replicates) were selected. Each block was divided into four plots, to which grazing treatments were randomized: no grazing (CK), sheep grazing (SG), yak grazing (YG), and mixed grazing by yak and sheep (MG). The grazing experiment was established to investigate how different livestock grazing affects ecosystem structure and function. Therefore, to avoid any factors that might confound the results, no activities such as mowing were performed in the CK plots. Similarly, in the experimental plots, livestock grazing was the only treatment factor and no other activities (e.g., mowing or fertilization) were performed.

**Figure 1:**
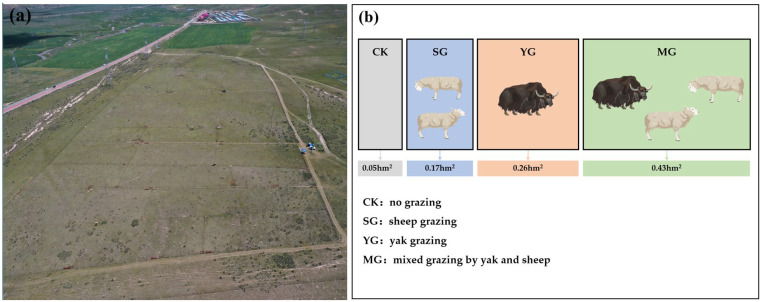
View of the study site (**a**); schematic diagram of the experimental design, including the number of yaks and sheep in each treatment and the area of the sample plots (**b**).

Based on their daily intake during the pre-trials (sheep: 1.2 ± 0.2 kg and yak: 3.7 ± 0.7 kg (mean ± SE)), we estimated that the grazing of three adult sheep is equivalent to that of one yak [33]. To ensure that there was equal grazing intensity among the different grazing treatments, the plot area was calculated based on the number of yaks and sheep. The details of livestock numbers and plot area are shown in Figure 1. Overall, the grazing intensity was maintained at a moderate intensity in each livestock treatment, which meant that half of the aboveground plant biomass was consumed by livestock. During the grazing treatment stage (from June to October), the grazing animals were kept in fenced experimental plots. They received no supplementary feeding but were given water every other day. Thus, the uneaten plants were not removed and the dung and urine were returned to the experimental plots. Before the onset of our sampling, the grazing experiment had been conducted continuously for 8 years.

### 2.3. Sampling

In August 2022, we randomly arranged three quadrats of 0.5 × 0.5 m^2^ in each of 12 grazing treatment plots and measured the severity of foliar fungal disease at the quadrat level. We recorded the disease severity on the leaves of each species of host plant in each quadrat and visually assessed the types of disease symptoms present, such as leaf spots, rust, and powdery mildew. We randomly selected 25 leaves per plant species, with five from the whole part of each of five randomly selected individuals per quadrat. For species with no more than five individuals per quadrat, we examined all the leaves. We defined disease severity as the percentage of leaf area that was covered by fungal lesions, which was estimated visually using cards with digital images of leaves of known disease severity [13,20]. We observed a total of 33 plant species across the 12 plots in our study. Out of these, 21 plant species were found to be infected with at least one fungal pathogen. In addition, we collected 10–20 samples of infected leaf tissue (with lesions) of each plant species, to confirm the pathogens in the laboratory using a light microscope (Stemi 305 Stereo Microscope, Zeiss, Jena, Germany). The pathogen taxonomy primarily followed the guidelines proposed by Dai (1979) and Braun and Cook (2012) [35,36], as well as previous studies of alpine meadows [2,37,38].

Simultaneously, we recorded the number of plant individuals (quantified based on the number of stems/ramets) of each species and the percentage coverage of each species and species richness (total number of species) per quadrat. We harvested the plant aboveground biomass of each species and dried the tissue at 70 °C for 48 h and then weighed it. We divided the plant species into four plant functional groups, including the *Cyperaceae*, forbs, grasses, and legumes. Furthermore, to test the relationships between plant disease and plant community characteristics, we calculated the Shannon–Weiner index (*H*′) and Pielou evenness index (*J_e_*) for each plot, as follows:

$$H^{\prime }=-\overset{n}{\underset{i=1}{\sum }}\left(\frac{n_{i}}{N}\mathrm{Ln}\frac{n_{i}}{N}\right)$$

$$J_{e}=\frac{H^{\prime }}{\mathrm{Ln}S}$$

where *n_i_* is the abundance of the *i*th plant species, *N* is the total abundance of plant species, and *S* is the total number of plant species in the plot.

### 2.4. Measures of Community-Level Indices

In each quadrat, we calculated the community pathogen load (*l*), as follows [20]:

$$l\;=\frac{\sum_{i=1}^{\;n}s_{i}c_{i}}{\sum_{i=1}^{n}c_{i}}$$

where *n* is the total number of plant species in the quadrat, *S_i_* is the severity index of the *i*th plant species, and *C_i_* is the percentage coverage of the *i*th species.

Previous studies showed that plant communities within the plots of different livestock treatments differed in composition [39,40], which could affect the types of foliar fungal diseases. Therefore, to test the magnitude to which the variation in community pathogen load could be explained by the variation in plant community composition, we calculated the prevalence of community disease. First, we defined a “disease proneness index” (*a_i_*) as the average disease severity of each species in the three control plots. We then calculated “community disease proneness” (*p*) for each plot by calculating a host percentage cover-weighted average of the ai total plant species per plot [41]:

$$p\;=\;\frac{\sum_{i=1}^{n}a_{i}c_{i}}{\sum_{i=1}^{n}c_{i}}$$

where *n* is the total number of plant species in the quadrat, *a_i_* is the disease proneness index of the *i*th plant species, and *C_i_* is the percentage coverage of the *i*th species.

### 2.5. Statistical Analyses

We used a one-way analysis of variance (ANOVA) with Tukey’s tests to analyze whether there were significant differences in the prevalence of foliar fungal diseases between the different grazing treatments. At the population level, we established the disease severity of individual species as the response variable and the grazing treatment as the independent variable. At the community level, we established the community pathogen load, pathogen load of different plant functional groups, and community disease severity as the response variables and the grazing treatment as the independent variable. We also established the disease severity of individual species and community pathogen load as the response variables, with plant community characteristics, including species richness (SR); Shannon–Weiner index (*H*′); Pielou’s evenness index (*J_e_*); plant aboveground biomass (PAB); and plant total coverage (PTC), as the independent variables to test their linear relationships. In addition, we used a random forest analysis to identify the importance of different plant species in influencing the community pathogen load, wherein the relative importance of all infested plant species was ranked. Before the ANOVA and linear models were constructed, the data were transformed (arcsine square roots or natural logarithms) when necessary to meet the model assumptions. All the statistical analyses were performed using R v. 3.5.1 [42].

## 3. Results

### 3.1. Population-Level Disease Severity

Of the 33 plant species observed in our experiment, 21 were infected with at least 1 foliar fungal disease (Table 1). Leaf spot diseases dominated the plant community and were observed in all the species of diseased plants (Table 1). One rust infection was identified on *Potentilla acaulis*, and one powdery mildew infection was identified on dandelion (*Taraxacum mongolicum*) (Table 1). Two dominant plant species, *K. humilis* and *C. aridula*, had the highest disease severity, followed by *Potentilla bifurca* and heartbroken grass (*Stellera chamaejasme*) (Table 1). The severity of eight leaf spot diseases decreased significantly with grazing by a single species or mixed species, whereas one leaf spot increased significantly in severity under grazing by a single species or mixed species (Table 1). The severity of three leaf spot diseases did not vary significantly with grazing by a single species or mixed species (Table 1). The severity of three leaf spot diseases decreased significantly only when grazed by yak (Table 1). In addition, two dominant plant species with the highest disease severity, *K. humilis* and *C. aridula*, decreased significantly under grazing by a single species or mixed species (Table 1).

### 3.2. Community Pathogen Load

**Figure 2:**
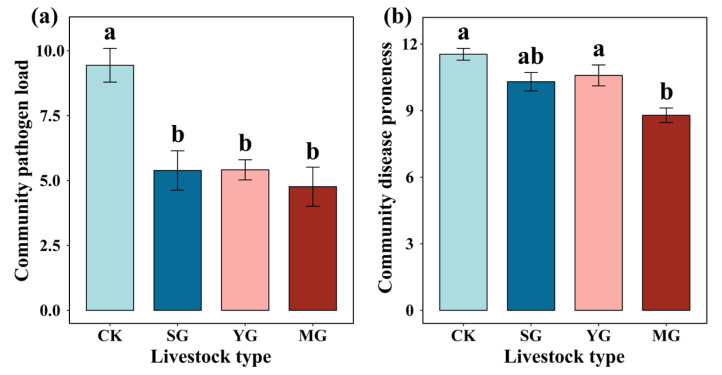
Effects of different livestock types on the community pathogen load (**a**) and community disease proneness (**b**). Different lowercase letters indicate significant differences between different livestock types at *p* < 0.05 (Tukey’s HSD). Error bars represent ±SE.

**Figure 3:**
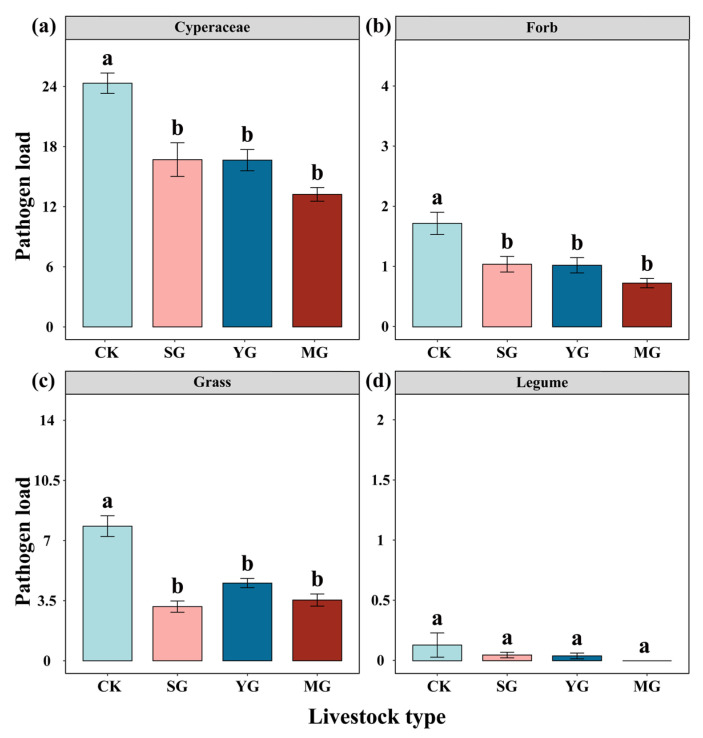
Effects of different livestock types on the pathogen load of different plant functional groups, i.e., *Cyperaceae* (**a**), forb (**b**), grass (**c**), and legume (**d**). Different lowercase letters indicate significant differences between different livestock types at *p* < 0.05 (Tukey’s HSD). Error bars represent ±SE.

**Figure 4:**
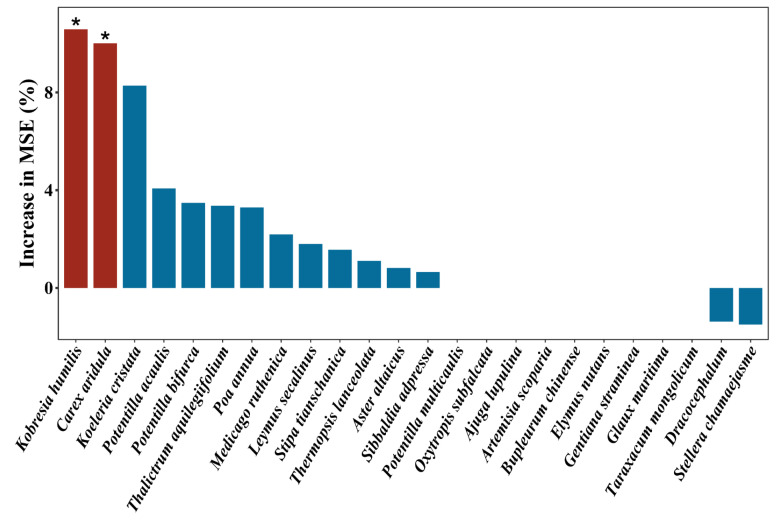
Relative importance of plant species disease severity in predicting community pathogen load based on random forest analysis (the percentage of increase in the mean variance error (MSE)). Significance levels: * 0.01 < *p* < 0.05.

At the plant community level, sheep grazing, yak grazing, and mixed grazing by both sheep and yak significantly decreased the community pathogen load (Figure 2a). Mixed grazing by both sheep and yak significantly decreased the community disease proneness (Figure 2b). However, we did not find a significant difference in community pathogen load among the different types of livestock (Figure 2a). In addition, sheep grazing, yak grazing, and mixed grazing by both types of livestock significantly decreased the pathogen load of *Cyperaceae*, forbs, and grasses (Figure 3a–c) but had no significant effect on the pathogen load of legumes (Figure 3d). The random forest model indicated that the most important predictor of community pathogen load was *K. humilis,* followed by *C. aridula* (*p* < 0.05) (Figure 4). Detailed data were showed in Table S1.

### 3.3. Plant Community Vegetation Characteristics

**Figure 5:**
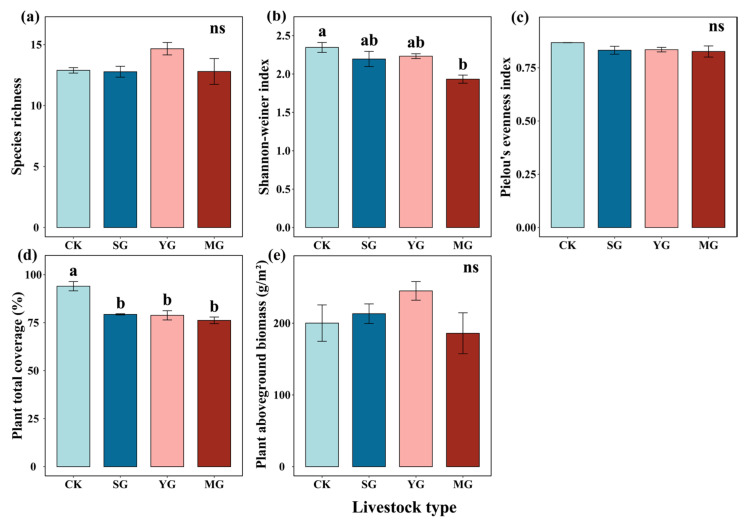
Effects of different livestock types on plant vegetation characteristics, i.e., species richness (**a**), Shannon–Weiner index (**b**), Pielou’s evenness index (**c**), plant total coverage (**d**), and plant aboveground biomass (**e**). ns indicates no significant difference; different lowercase letters indicate significant differences between different livestock types at *p* < 0.05 (Tukey’s HSD). Error bars represent ±SE.

Sheep grazing, yak grazing, and mixed grazing by both sheep and yak significantly decreased the plant total coverage (Figure 5d). Mixed grazing by both sheep and yak significantly decreased the Shannon–Weiner index (Figure 5b). However, grazing by a single species or mixed species did not significantly affect the plant species richness, Pielou’s evenness index, or plant aboveground biomass (Figure 5a,c,e). Detailed data were showed in Table S2.

### 3.4. Environmental Factors That Influenced Foliar Fungal Diseases

**Figure 6:**
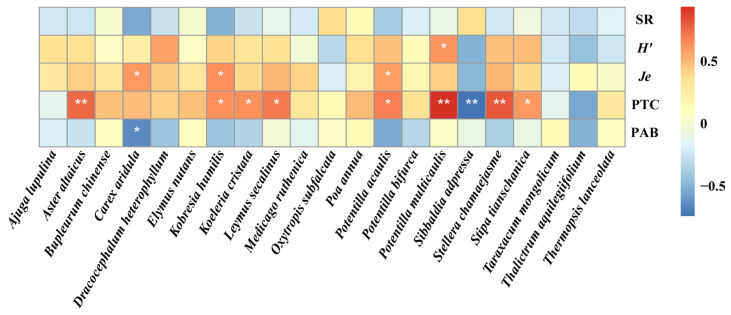
Relationships between disease severity of individual species and plant community characteristics (PAB, plant aboveground biomass; PTC, plant total coverage; *Je*, Pielou’s evenness index; *H*′, Shannon–Weiner index; SR, species richness). Blue indicates a negative correlation, red indicates a positive correlation, and the depth of the color represents the magnitude of the correlation coefficient. Asterisks indicate statistical significance (* *p* < 0.05; ** *p* < 0.01).

**Figure 7:**
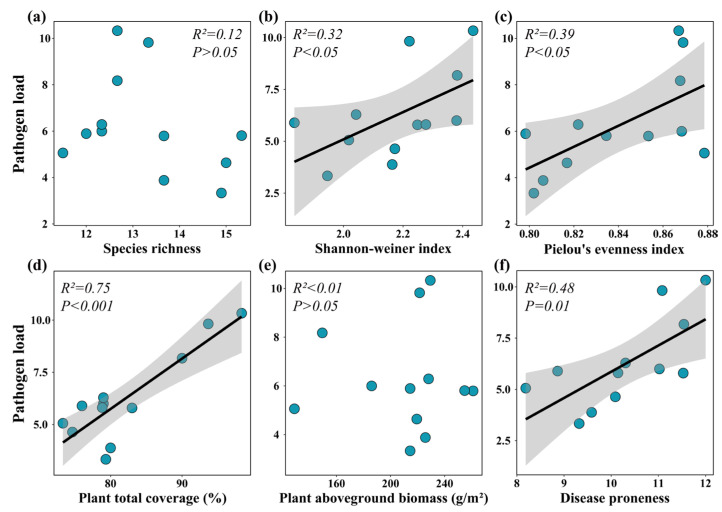
Relationships between community pathogen load and species richness (**a**), Shannon–Weiner index (**b**), Pielou’s evenness index (**c**), plant total coverage (**d**), plant aboveground biomass (**e**), and community disease proneness (**f**). The black fitted regression lines indicate significant relationships at the level of *p* < 0.05, with 95% confidence intervals shown as shaded areas. The coefficient of determination (*R*^2^) and *p* values are shown.

At the population level, the disease severity of eight plant species positively correlated with the total plant coverage, and the disease severity of one plant species negatively correlated with the total plant coverage (Figure 6). The disease severity of three plant species positively correlated with Pielou’s evenness index (Figure 6). The disease severity of one plant species positively correlated with the Shannon–Weiner index (Figure 6). Similarly, the disease severity of one plant species negatively correlated with the plant aboveground biomass (Figure 6). At the plant community level, the community pathogen load positively correlated with the Shannon’s diversity index, Pielou’s evenness index, total plant coverage, and community disease severity (Figure 7b–d,f). However, there were no significant relationships between community pathogen load and species richness and plant aboveground biomass (Figure 7a,e).

## 4. Discussion

Our study revealed that grazing by a single species (yak or sheep) or mixed species (sheep and yak) consistently decreased the occurrence of foliar fungal diseases, which supported our first hypothesis. Previous studies noted that the reduction in foliar fungal diseases following grazing is largely owing to the presence of biotrophic diseases, such as rusts and powdery mildew that can only survive in living plants and rely on transmission by spores [43]. Thus, grazing on leaves infected with powdery mildew or rust can remove the spores, thereby decreasing the transmission of disease [13,44]. In contrast, the pathogens of necrotrophic diseases, primarily leaf spots, can survive in various reservoirs such as soil and plant residue, enabling them to colonize plant tissues through the wounds produced by grazers [17,45,46,47]. However, in our study system, leaf spots dominated the plant community, and the reduction in foliar fungal diseases was primarily ascribed to a decrease in leaf spots. We found that the disease severity of eight leaf spot diseases decreased significantly under grazing disturbance at the population level. Similarly, at the community level, grazing by a single species (yak or sheep) or mixed species (sheep and yak) significantly decreased the community pathogen load. These findings contrast with several previous studies that showed that grazing had positive or neutral effects on leaf spots [13]. One possible explanation is that the wounds caused by grazing may have induced leaf tissues to quickly activate their defense responses, resulting in enhanced resistance to necrotrophic pathogens, since plant defenses to various attackers, such as herbivores or diseases, are usually synergistic [14,48]. In another experiment with bitter dock (*Rumex obtusifolius*), beetle (*Gastrophysa viridula*) grazing was found to increase the number of lesions caused by the necrotrophic fungal pathogen *Ramularia rubella* compared with the controls, which provides further evidence for this hypothesis [49].

We hypothesized that different livestock may differentially affect foliar fungal diseases because of differences in their grazing behaviors (e.g., diet selectivity) and livestock characteristics (e.g., body size) [29,50]. However, our study showed that grazing by yak did not lead to more pronounced reductions in the occurrence of foliar fungal diseases than grazing by sheep, which did not support our second hypothesis. This could likely be attributed to the fact that different livestock vary in their feeding preferences. For example, large livestock usually feed on tall grasses, whereas small livestock prefer low-growing forbs and semi-shrubs. Thus, long-term intensive grazing may largely consume plant aboveground material and can remove 50% of the aboveground biomass; thus, largely removing pathogens through defoliation [28,29,30,44]. Another possible explanation could relate to grassland type. A recent study in a semi-arid grassland found that 13 years of grazing by sheep indirectly increased the community pathogen load, by increasing the abundances of grazing-tolerant hosts [13]. Compared with semi-arid grasslands, alpine grasslands generally have higher vegetation cover and plant species diversity, and their plant community structures are more resistant to grazing disturbances [51,52]. In our study system (an alpine grassland), we found that 8 years of grazing by either yak or sheep did not significantly alter the plant species richness and Shannon diversity. Therefore, we hypothesized that in alpine grasslands with diversified plant communities, the indirect effects of grazing on foliar fungal diseases through changes in plant community characteristics are limited.

Another intriguing finding was that two species of *Cyperaceae* (*C. aridula* and *K. humilis*) experienced higher disease severity than other species in our study system, probably because *C. aridula* and *K. humilis* are both more palatable and susceptible to foliar fungal pathogens [53,54]. Our random forest model also showed that the disease severity of *C. aridula* and *K. humilis* was the most important predictor of community pathogen load, which suggests that the two species play a key role in driving the occurrence and transmission of foliar fungal diseases in the plant community. The density-dependent transmission of foliar fungal diseases could be a potential mechanism that underlies the higher disease severity observed in *C. aridula* and *K. humilis,* since the two species were the dominant species in our study site [55]. An increase in the occurrence of foliar fungal diseases in dominant species can inhibit the expansion of the dominance of their host plants in the community through negative density dependence, thereby facilitating the coexistence of species [3,56,57]. In addition, previous studies indicated that increased host species richness can exert a dilution effect on disease transmission and prevalence [20,21]. In our study, we did not find negative correlations between species richness and the community pathogen load, suggesting that grazing can reverse the relationship between species richness and disease transmission, leading to the disappearance of the dilution effect.

While our study showed that grazing by livestock decreased the occurrence of foliar fungal diseases, the following aspects could potentially have biased our conclusions. First, the microenvironment, such as temperature and humidity, are important factors that influence spore production and mycelial growth and thus the occurrence and transmission of diseases [58,59]. There is some evidence that grazing can increase the temperature but decrease the humidity of the vegetation microenvironment. For example, this was observed in a study of a temperate meadow [25]. Second, the supply of nutrients can affect the transmission and prevalence of disease [19,60]. For example, a high supply of nitrogen can increase disease severity by promoting the growth of fungal pathogens or by leading the community composition toward more disease-prone plant species [19]. Many experimental studies and meta-analyses have indicated that grazing can alter the chemistry of leaves [61,62], and thus may indirectly affect foliar fungal diseases. Moreover, the responses of plant defense systems to grazing and fungal infection are synergistic, yet still different [14,48]. For example, jasmonates are primarily involved in defense against herbivores, whereas salicylic acid is primarily associated with defense against pathogen infections [14,48]. Livestock grazing can stimulate plant defense systems, but how this response affects the ability to defend against foliar fungal pathogens remains unclear [63]. Therefore, considering that grazing can alter the microenvironment, plant nutrient supply and defense systems, the measurement of environmental, nutritional, and defensive parameters is necessary to fully understand how foliar fungal diseases respond to livestock grazing through different pathways.

In conclusion, our study indicated that grazing by a single species (yak or sheep) or mixed species (sheep and yak) consistently decreased the occurrence of foliar fungal diseases. However, the reductions in foliar fungal diseases caused by grazing did not vary with the type of livestock. To the best of our knowledge, this is the first study to have explored how different livestock species affect foliar fungal diseases. These findings not only advance our understanding of the interface between disease ecology and large herbivores under increasing human activities, but also provide some insights into the management of alpine grasslands. Considering that the intensity of grazing in each livestock treatment was maintained at a moderate intensity, we suggest that moderate grazing by sheep or yak is an effective way to control the occurrence of foliar fungal diseases in alpine grasslands.

## Figures and Tables

**Table 1:** List of 21 infected plant species and their disease types, associated pathogens, and disease severity (mean ± SE) with different livestock type treatments. CK, control; SG, sheep grazing alone; YG, yak grazing alone; MG, mixed grazing by yak and sheep.

Plant Species	Functional Group	Disease Types	Associated Pathogens	Livestock Types				Mean Disease Severity
				CK	SG	YG	MG	
*Ajuga lupulina*	Forb	Leaf spot	Undentified	NA	NA	10 *	NA	NA
*Aster altaicus*	Forb	Leaf spot	Undentified	5.3 ± 1.0	0.7 ± 0.7	0.00 *	0.00 *	NA
*Bupleurum chinense*	Forb	Leaf spot	*Septoria* sp.; …	37.6 *	NA	NA	NA	NA
*Carex aridula*	*Cyperaceae*	Leaf spot	*Alternaria* sp.; …	38.9 ± 3.0 a	30.7 ± 6.9 b	27.6 ± 1.8 b	20.4 ± 1.2 b	29.4 ± 3.2
*Dracocephalum heterophyllum*	Forb	Leaf spot	*Alternaria solani*; …	11.9 ± 1.5 a	6.7 ± 1.3 b	9.0 ± 3.3 a	5.7 ± 2.0 b	8.3 ± 1.4
*Elymus nutans*	Grass	Leaf spot	Undentified	18.4 *	NA	NA	NA	NA
*Kobresia humilis*	*Cyperaceae*	Leaf spot	*Alternaria alternata*; …	42.1 ± 2.1 a	25.0 ± 6.6 b	27.8 ± 2.4 b	23.7 ± 2.0 b	29.7 ± 3.5
*Koeleria cristata*	Grass	Leaf spot	Undentified	28.2 ± 1.2 a	13.9 ± 2.4 b	12.4 ± 1.5 b	10.4 ± 4.8 b	16.2 ± 3.0
*Leymus secalinus*	Grass	Leaf spot	*Coniochaeta discospora*; …	23.8 ± 6.7 a	8.4 ± 0.9 b	11.2 ± 1.8 b	9.3 ± 1.1 b	13.2 ± 2.9
*Medicago ruthenica*	Legume	Leaf spot	Undentified	22.40 *	3.2 ± 1.8	1.9 ± 1.5	NA	NA
*Oxytropis subfalcata*	Legume	Leaf spot	Undentified	0.4 ± 0.4 a	0.7 ± 0.7 a	0.0 ± 0.0 a	0.0 ± 0.0 a	0.3 ± 0.2
*Poa annua*	Grass	Leaf spot	*Phoma* sp.; …	17.5 ± 5.1 a	5.9 ± 0.7 b	13.8 ± 0.4 ab	9.3 ± 3.8 b	11.6 ± 2.3
*Potentilla acaulis*	Forb	Leaf spot	Undentified	27.7 ± 4.1 a	14.4 ± 3.2 b	12.6 ± 1.0 b	9.6 ± 0.8 b	16.1 ± 2.9
		Rust	*Phragmidium potentillae*; …	NA	NA	3.2 *	NA	NA
*Potentilla bifurca*	Forb	Leaf spot	*Coniochaeta* sp.; …	37.2 ± 7.5 a	11.2 ± 2.4 b	15.1 ± 2.9 b	10.4 ± 5.2 b	17.4 ± 4.3
*Potentilla multicaulis*	Forb	Leaf spot	*Stagonospora* sp.; …	25.6 ± 3.9 a	5.6 ± 2.1 b	10.8 ± 0.9 b	6.9 ± 1.6 b	12.2 ± 3.2
*Sibbaldia adpressa*	Forb	Leaf spot	*Leptotrochila* sp.; …	7.2 ± 0.8 b	16.3 ± 3.0 a	14.6 ± 1.4 a	13.3 ± 1.0 a	12.9 ± 1.6
*Stellera chamaejasme*	Forb	Leaf spot	*Acrophialophora jodhpurensis*; …	33.6 ± 2.4 a	13.9 ± 0.6 b	11.7 ± 2.5 b	9.7 ± 0.8 b	17.2 ± 3.7
*Stipa tianschanica*	Grass	Leaf spot	*Septora* sp.; …	20.5 ± 4.3 a	8.0 ± 0.5 b	14.2 ± 1.4 ab	11.9 ± 1.2 b	13.7 ± 2.1
*Taraxacum mongolicum*	Forb	Powdery mildew	*Sphaerotheca fusca*; …	NA	NA	56.7 *	NA	NA
*Thalictrum aquilegiifolium*	Forb	Leaf spot	*Alternaria alternata*; …	NA	20.8 ± 13.4	5.2 ± 3.7	8.6 ± 0.8	NA
*Thermopsis lanceolata*	Legume	Leaf spot	*Cercospora* sp.; …	4.0 *	2.4 *	NA	NA	NA

Notes: NA indicates missing values; * indicates standard error (SE) cannot be calculated for this value (i.e., sufficient degrees of freedom); different lowercase letters indicate significant differences between livestock types at *p* < 0.05 (Tukey’s HSD). “…” indicates other possible pathogens.

## Data Availability

All applicable data are published and referenced in the paper.

## Supplementary Materials

The following supporting information can be downloaded at: https://www.mdpi.com/article/10.3390/jof9090949/s1, Table S1: Pathogen load [community pathogen load (*l*); *Cyperaceae* pathogen load (C*l*); Forb pathogen load (F*l*); Grass pathogen load (G*l*); Legume pathogen load (L*l*)] and community disease proneness (*p*) (g; mean ± SE) under no grazing (CK), sheep grazing (SG), yak grazing (YG), and mixed grazing by yak and sheep (MG) treatments; Table S2: Plant community characteristics [Shannon-weiner index (*H’*); Pielou’s evenness index (*Je*); species richness (SR); plant total coverage (PTC); plant aboveground biomass (PAB)] (g; mean ± SE) under no grazing (CK), sheep grazing (SG) yak grazing (YG), and mixed grazing by yak and sheep (MG) treatments.

## References

[1] Fisher M.C., Henk D.A., Briggs C.J., Brownstein J.S., Madoff L.C., McCraw S.L., Gurr S.J. (2012). Emerging fungal threats to animal, plant and ecosystem health. Nature.

[2] Liu X., Ma Z., Cadotte M.W., Chen F., He J., Zhou S. (2019). Warming affects foliar fungal diseases more than precipitation in a Tibetan alpine meadow. New Phytol..

[3] Chen L., Swenson N.G., Ji N., Mi X., Ren H., Guo L., Ma K. (2019). Differential soil fungus accumulation and density dependence of trees in a subtropical forest. Science.

[4] Maron J.L., Marler M., Klironomos J.N., Cleveland C.C. (2011). Soil fungal pathogens and the relationship between plant diversity and productivity. Ecol. Lett..

[5] Bagchi R., Gallery R.E., Gripenberg S., Gurr S.J., Narayan L., Addis C.E., Freckleton R.P., Lewis O.T. (2014). Pathogens and insect herbivores drive rainforest plant diversity and composition. Nature.

[6] Cappelli S.L., Pichon N.A., Kempel A., Allan E. (2020). Sick plants in grassland communities: A growth-defense trade-off is the main driver of fungal pathogen abundance and impact. Ecol. Lett..

[7] Seabloom E.W., Kinkel L., Borer E.T., Hautier Y., Montgomery R.A., Tilman D. (2017). Food webs obscure the strength of plant diversity effects on primary productivity. Ecol. Lett..

[8] Bever J.D., Mangan S.A., Alexander H.M. (2015). Maintenance of plant species diversity by pathogens. Annu. Rev. Ecol. Evol. Syst..

[9] Parker I.M., Saunders M., Bontrager M., Weitz A.P., Hendricks R., Magarey R., Suiter K., Gilbert G.S. (2015). Phylogenetic structure and host abundance drive disease pressure in communities. Nature.

[10] Milchunas D.G., Sala O.E., Lauenroth W.K. (1988). A generalized model of the effects of grazing by large herbivores on grassland community structure. Am. Nat..

[11] Filazzola A., Brown C., Dettlaff M.A., Batbaatar A., Grenke J., Bao T., Heida I.P., Cahill J. (2020). The effects of livestock grazing on biodiversity are multi-trophic: A meta-analysis. Ecol. Lett..

[12] Koerner S.E., Smith M.D., Burkepile D.E., Hanan N.P., Avolio M.L., Collins S.L., Knapp A.K., Lemoine N.P., Forrestel E.J., Eby S. (2018). Change in dominance determines herbivore effects on plant biodiversity. Nat. Ecol. Evol..

[13] Liu Y., Duan D., Jiang F., Tian Z., Feng X., Wu N., Hou F., Kardol P., Nan Z., Chen T. (2021). Long-term heavy grazing increases community-level foliar fungal diseases by shifting plant composition. J. Appl. Ecol..

[14] Stout M.J., Thaler J.S., Thomma B.P.H.J. (2006). Plant-mediated interactions between pathogenic microorganisms and herbivorous arthropods. Annu. Rev. Entomol..

[15] Borer E.T., Mitchell C.E., Power A.G., Seabloom E.W. (2009). Consumers indirectly increase infection risk in grassland food webs. Proc. Natl. Acad. Sci. USA.

[16] Packer C., Holt R.D., Hudson P.J., Lafferty K.D., Dobson A.P. (2003). Keeping the herds healthy and alert: Implications of predator control for infectious disease. Ecol. Lett..

[17] Daleo P., Silliman B., Alberti J., Escapa M., Canepuccia A., Peña N., Iribarne O. (2009). Grazer facilitation of fungal infection and the control of plant growth in south-western Atlantic salt marshes. J. Ecol..

[18] Skipp R.A., Lambert M.G. (1984). Damage to white clover foliage in grazed pastures caused by fungi and other organisms. N. Z. J. Agric. Res..

[19] Liu X., Lyu S., Sun D., Bradshaw C., Zhou S. (2017). Species decline under nitrogen fertilization increases community-level competence of fungal diseases. Proc. R. Soc. B.

[20] Mitchell C., Tilman D., Groth J. (2002). Effects of grassland plant species diversity, abundance, and composition on foliar fungal disease. Ecology.

[21] Rottstock T., Joshi J., Kummer V., Fischer M. (2014). Higher plant diversity promotes higher diversity of fungal pathogens, while it decreases pathogen infection per plant. Ecology.

[22] Ostfeld R., Holt R. (2004). Are predators good for your health? Evaluating evidence for top-down regulation of zoonotic disease reservoirs. Front. Ecol. Environ..

[23] Herrero-Jáuregui C., Oesterheld M. (2018). Effects of grazing intensity on plant richness and diversity: A meta-analysis. Oikos.

[24] Schönbach P., Wan H., Gierus M., Bai Y., Müller K., Lin L., Susenbeth A., Taube F. (2011). Grassland responses to grazing: Effects of grazing intensity and management system in an Inner Mongolian steppe ecosystem. Plant Soil.

[25] Zhang Y., Nan Z., Xin X. (2020). Response of plant fungal diseases to beef cattle grazing intensity in Hulunber grassland. Plant Dis..

[26] Mipam T.D., Chen F., Tian L., Zhang P., Huang M., Chen L., Wang X., Zhang P., Lin Z., Liu X. (2022). Plant community-mediated effects of grazing on plant diseases. Oecologia.

[27] Su J., Xu F. (2021). Root, not aboveground litter, controls soil carbon storage under grazing exclusion across grasslands worldwide. Land Degrad. Dev..

[28] Dumont B., Carrère P., Ginane C., Farruggia A., Lanore L., Tardif A., Decuq F., Darsonville O., Louault F. (2011). Plant-herbivore interactions affect the initial direction of community changes in an ecosystem manipulation experiment. Basic Appl. Ecol..

[29] Tóth E., Deák B., Valkó O., Kelemen A., Miglécz T., Tóthmérész B., Török P. (2018). Livestock type is more crucial than grazing intensity: Traditional cattle and sheep grazing in short-grass steppes. Land Degrad. Dev..

[30] Eldridge D.J., Ding J., Travers S.K. (2022). A global synthesis of the effects of livestock activity on hydrological processes. Ecosystems.

[31] Crawley M.J. (1983). Herbivory: The Dynamics of Plant-Animal Interactions.

[32] Gao J., Carmel Y. (2020). Can the intermediate disturbance hypothesis explain grazing-diversity relations at a global scale?. Oikos.

[33] Yang X., Dong Q., Chu H., Ding C., Yu Y., Zhang C., Zhang Y., Yang Z. (2019). Different responses of soil element contents and their stoichiometry (C:N:P) to yak grazing and Tibetan sheep grazing in an alpine grassland on the eastern Qinghai-Tibetan Plateau. Agric. Ecosyst. Environ..

[34] Zhang M., Zhao L., Hu J., Khan A., Yang X., Dong Q., Rensing R., Fang X., Zhang J. (2023). Different grazers and grazing practices alter the growth, soil properties, and rhizosphere soil bacterial communities of *Medicago ruthenica* in the Qinghai-Tibetan Plateau grassland. Agric. Ecosyst. Environ..

[35] Dai F. (1979). Sylloge Fungorum Sinicorum.

[36] Braun U., Cook R. (2012). Taxonomic Manual of the Erysiphales (Powdery Mildews).

[37] Zhang R. (2009). Survey and Identification of the Alpine Grassland’s Major Fungal Diseases in Gannan Region of Gansu Province. Master’s Thesis.

[38] Liu X., Lyu S., Zhou S., Bradshaw C.J. (2016). Warming and fertilization alter the dilution effect of host diversity on disease severity. Ecology.

[39] Wang L., Delgado-Baquerizo M., Wang D., Isbell F., Liu J., Feng C., Liu J., Zhong Z., Zhu H., Yuan X. (2019). Diversifying livestock promotes multidiversity and multifunctionality in managed grasslands. Proc. Natl. Acad. Sci. USA.

[40] Liu J., Feng C., Wang D., Wang L., Wilsey B.J., Zhong Z. (2015). Impacts of grazing by different large herbivores in grassland depend on plant species diversity. J. Appl. Ecol..

[41] Mitchell C.E., Reich P.B., Tilman D., Groth J.V. (2003). Effects of elevated CO_2_, nitrogen deposition, and decreased species diversity on foliar fungal plant disease. Glob. Chang. Biol..

[42] R Development Core Team (2016). R: A Language and Environment for Statistical Computing. R Foundation for Statistical Computing. http://www.r-project.org/.

[43] Gilbert G.S., Parker I.M. (2016). The evolutionary ecology of plant disease: A phylogenetic perspective. Annu. Rev. Phytopathol..

[44] Zhang Y., Nan Z., Christensen M.J., Xin X., Zhang N. (2022). Effects of rust on plant growth and stoichiometry of *Leymus chinensis* under different grazing intensities in Hulunber grassland. Agriculture.

[45] Tedersoo L., Bahram M., Põlme S., Kõljalg U., Yorou N.S., Wijesundera R., Ruiz L.V., Vasco-Palacios A.M., Thu P.Q., Suija A. (2014). Global diversity and geography of soil fungi. Science.

[46] Delgado-Baquerizo M., Guerra C.A., Cano-Díaz C., Egidi E., Wang J., Eisenhauer N., Singh B.K., Maestre F.T. (2020). The proportion of soil-borne pathogens increases with warming at the global scale. Nat. Clim. Chang..

[47] Liu M., Mipam T.D., Wang X., Zhang P., Lin Z., Liu X. (2021). Contrasting effects of mammal grazing on foliar fungal diseases: Patterns and potential mechanisms. New Phytol..

[48] Moreira X., Abdala-Roberts L., Castagneyrol B. (2018). Interactions between plant defence signalling pathways: Evidence from bioassays with insect herbivores and plant pathogens. J. Ecol..

[49] Hatcher P.E., Paul N.D. (2000). Beetle grazing reduces natural infection of *Rumex obtusifolius* by fungal pathogens. New Phytol..

[50] Bremm C., Laca E.A., Fonseca L., Mezzalira J.C., Elejalde D.A.G., Gonda H.L., de Faccio Carvalho P.C. (2012). Foraging behaviour of beef heifers and ewes in natural grasslands with distinct proportions of tussocks. Appl. Anim. Behav. Sci..

[51] MacArthur R. (1955). Fluctuations of animal populations and a measure of community stability. Ecology.

[52] Tilman D., Isbell F., Cowles J.M. (2014). Biodiversity and ecosystem functioning. Annual Review of Ecology. J. Syst. Evol..

[53] Wang N., Zhang Z., Zhou H., Xu W. (2022). Effects of temperature, cold stratification and chemical treatment on seed germination of three Cyperaceae species. Acta Agrestia Sin..

[54] Liu X., Lu Y., Zhang Z., Zhou S. (2020). Foliar fungal diseases respond differently to nitrogen and phosphorus additions in Tibetan alpine meadows. Ecol. Res..

[55] Dong Q., Zhao X., Liu Y., Feng B., Yu Y., Yang X., Zhang C., Cao C., Liu W. (2022). Effects of different herbivore assemblage on relationship between *Kobresia humilis* seed size and seed number in an alpine grassland. Chin. J. Ecol..

[56] LaManna J.A., Mangan S.A., Alonso A., Bourg N.A., Brockelman W.Y., Bunyavejchewin S., Chang L.W., Chiang J.M., Chuyong G.B., Clay K. (2017). Plant diversity increases with the strength of negative density dependence at the global scale. Science.

[57] Bagchi R., Swinfield T., Gallery R.E., Lewis O.T., Gripenberg S., Narayan L., Freckleton R.P. (2010). Testing the Janzen-Connell mechanism: Pathogens cause overcompensating density dependence in a tropical tree. Ecol. Lett..

[58] Helfer S. (2014). Rust fungi and global change. New Phytol..

[59] Launay M., Caubel J., Bourgeois G., Huard F., de Cortazar-Atauri I.G., Bancal M.O., Brisson N. (2014). Climatic indicators for crop infection risk: Application to climate change impacts on five major foliar fungal diseases in Northern France. Agric. Ecosyst. Environ..

[60] Borer E.T., Seabloom E.W., Mitchell C.E., Cronin J.P. (2014). Multiple nutrients and herbivores interact to govern diversity, productivity, composition, and infection in a successional grassland. Oikos.

[61] Bai Y., Wu J., Clark C.M., Pan Q., Zhang L., Chen S., Wang Q., Han X. (2012). Grazing alters ecosystem functioning and C:N:P stoichiometry of grasslands along a regional precipitation gradient. J. Appl. Ecol..

[62] Ritchie M.E., Tilman D., Knops J.M.H. (2008). Herbivore effects on plant and nitrogen dynamics in oak savanna. Ecology.

[63] Jonas J.L., Joern A. (2008). Host-plant quality alters grass/forb consumption by a mixed-feeding insect herbivore, *Melanoplus bivittatus* (Orthoptera: Acrididae). Ecol. Entomol..

