# Supplementary material for: Effects of Different Livestock Grazing on Foliar Fungal Diseases in an Alpine Grassland on the Qinghai–Tibet Plateau
Source: J Fungi (Basel). 2023 Sep 20;9(9):949. doi: 10.3390/jof9090949 (PMC10533196; doi:10.3390/jof9090949)
Supplement: Supplementary file 1 [file jof-09-00949-s001.zip › jof-2566940-supplementary.pdf]

Supporting Information

Table S1. Pathogen load [community pathogen load (*l*); *Cyperaceae* pathogen load (C*l*); Forb pathogen load (F*l*); Grass pathogen load (G*l*); Legume pathogen load (L*l*)] and community disease proneness (*p*) (g; mean ± SE) under no grazing (CK), sheep grazing (SG), yak grazing (YG), and mixed grazing by yak and sheep (MG) treatments.

Grazing treatment	*l*	*p*	C*l*	F*l*	G*l*	L*l*
CK	9.4±0.6	11.5±0.3	24.3±1.0	1.7±0.2	7.8±0.6	0.1±0.1
SG	5.4±0.8	10.3±0.4	16.7±1.7	1.1±0.1	3.2±0.3	0.04±0.02
YG	5.4±0.4	10.6±0.5	16.6±1.1	1.0±0.1	4.5±0.3	0.03±0.02
MG	4.8±0.8	8.8±0.3	13.2±0.7	0.8±0.1	3.5±0.4	0.00±0.00

Table S2. Plant community characteristics [Shannon-weiner index (*H'*); Pielou's evenness index (*Je*); species richness (SR); plant total coverage (PTC); plant aboveground biomass (PAB)] (g; mean ± SE) under no grazing (CK), sheep grazing (SG) yak grazing (YG), and mixed grazing by yak and sheep (MG) treatments.

Grazing treatment	*H'*	*Je*	SR	PTC	PAB
CK	2.3±0.1	0.9±0.0	12.9±0.2	94.0±2.0	200.1±25.4
SG	2.2±0.1	0.8±0.0	12.8±0.4	79.3±0.3	213.3±13.7
YG	2.2±0.0	0.8±0.0	14.7±0.5	78.8±2.4	245.1±13.0
MG	1.9±0.1	0.8±0.1	12.8±1.1	76.2±1.7	186.0±28.5
